# 2464 Sexual dimorphism in a mouse model of syndromic thoracic aortic aneurysm

**DOI:** 10.1017/cts.2018.121

**Published:** 2018-11-21

**Authors:** Zheying Chen, Alan Daugherty, Mary Sheppard

**Affiliations:** 1 Saha Cardiovascular Research Center, University of Kentucky

## Abstract

OBJECTIVES/SPECIFIC AIMS: Pre-clinical and clinical observations have noted that increased aortic dilation is associated with male sex. Using an experimental model of severe, syndromic thoracic aortic aneurysms, we quantify aortic dilation and elastin stability in male Versus female mice. METHODS/STUDY POPULATION: Ascending aortas from male and female FBN1mgR/mgR mice and their wild type littermates were assessed every 4 weeks from 6 to 18 weeks of age by ultrasound. Measurements were taken luminal edge to luminal edge in diastole. At termination, aortas were harvested for RT-PCR analysis of extracellular matrix genes. Aortas were serially sectioned and elastin fragmentation was imaged by auto-fluorescence. RESULTS/ANTICIPATED RESULTS: At 12 weeks of age, differences of aortic diameters between male and female FBN1mgR/mgR mice were significantly different (2.24±0.43 vs. 1.57±0.22 mm; *p*=0.002), while there were no significant differences between sexes of wild type littermates (1.29±0.13 vs. 1.23±0.08 mm; *p*=0.71). Male sex was associated with increased elastin but not fibrillin-1 mRNA expression. Ascending aortas from male and female FBN1mgR/mgR mice significantly differed in the degree of elastin fragmentation (2.76 vs. 1.85 breaks/ 100 µm aorta; *p*=0.03). DISCUSSION/SIGNIFICANCE OF IMPACT: Sexual dimorphism of thoracic aortic dilation observed in human TAA patients was recapitulated in the fibirllin-1 hypomorphic mouse model of syndromic thoracic aortic aneurysms. Differences in this mouse model could be explained by the differential expression of extracellular matrix genes.

